# Vaginal pixel CO2 laser versus topical use of promestriene for genitourinary syndrome of menopause

**DOI:** 10.61622/rbgo/2026rbgo13

**Published:** 2026-02-20

**Authors:** Kátia Beckhauser, Marina Gules Bernardi, Edison Capp, Lúcia Maria Kliemann, Maria Celeste Osório Wender

**Affiliations:** 1 Universidade Federal do Rio Grande do Sul Porto Alegre RS Brazil Universidade Federal do Rio Grande do Sul, Porto Alegre, RS, Brazil.

**Keywords:** CO_2_ laser, Promestriene, Vulvovaginal atrophy, Urinary incontinence, Menopause, Visual Analog Scale Neutrophils, Lasers, gas, Orgasm, Personal satisfaction

## Abstract

**Objective:**

To compare the effectiveness of pixel CO_2_ laser and vaginal promestriene in treating genitourinary syndrome of menopause (GSM).

**Methods:**

A quasi-randomized controlled trial was conducted with 48 patients. CO_2_ Laser Group (24 patients) received 3 sessions of vaginal pixel CO_2_ laser, and promestriene group (24 patients) used vaginal promestriene daily for 14 days, then twice weekly for 3 months and 3 weeks. Patients were evaluated before and after treatment using a visual analog scale (VAS), FSFI-6, ICIQ-SF, Vaginal Health Index (VHI), and vaginal wall biopsy.

**Results:**

Of the 48 patients, 22 in CO_2_ Laser Group and 21 in promestriene group completed the study. Both groups showed significant symptom reduction by VAS, with improvements in desire, lubrication, and total FSFI-6 scores. CO_2_ Laser Group had greater improvements in lubrication, orgasm, and satisfaction (p<0.001). Urinary incontinence improved in both groups (p<0.01). VHI scores increased significantly in both groups (p<0.001). Biopsies revealed improvements in collagen, glycogen, vascularity, epithelial thickness, and reduced neutrophil count (p<0.01).

**Conclusion:**

CO_2_ laser appears to be a viable non-hormonal alternative for treating GSM, particularly for women unable or unwilling to use hormonal therapies.

## Introduction

By 2025, approximately one billion women will be in menopause, according to the World Health Organization (WHO) and up to 50% may suffer from symptoms related to genitourinary syndrome of menopause (GSM).^(1-3)^

Vaginal treatments such as conjugated estrogen, estradiol, estriol, and promestriene, have proven to be effective and are the preferred options for alleviating the symptoms of GSM. However, topical hormone therapy (HT) is based on daily or weekly application; its effect is dependent upon regular use, and if discontinued, the symptoms quickly return.^(4)^ In addition, not all women are candidates for HT and have no other option than to live with the symptoms, use symptomatic treatments that are often ineffective, and deprive themselves of a better quality of life.

In this context, vaginal laser therapy appears to be a non-hormonal treatment option, which is applied directly to the tissues, increasing the temperature, stimulating angiogenesis, production of collagen/elastin, restoring lubrication and vascularization of the vaginal mucosa, and reducing the symptoms of vaginal atrophy.^(5-7)^

The first vaginal CO_2_ laser probes were launched in 2013 and since then several studies have been published to confirm the effectiveness of this method. However, many of these studies required better-defined protocols, a control group, and an adequate sample.^(8)^ In addition, few studies use histopathology, through biopsy of the vagina, as a parameter to evaluate the effectiveness of the laser, which could definitively prove its effect on the vaginal wall. Therefore, this study aimed to compare the use of the vaginal pixel CO_2_ laser with the use of vaginal promestriene in the treatment of GSM based on the following 5 variables: Visual Analogue Scale (VAS) for GSM symptoms, Questionnaire on Sexual Functioning and Quality of Life (FSFI-6), Questionnaire for the Evaluation of Urinary Incontinence [International Consultation on Incontinence Questionnaire-Short Form (ICIQ-SF)], Vaginal Health Index Score (VHI) and histopathology of vaginal mucosa biopsy.

## Methods

A quasi-randomized controlled trial (participants were allocated to treatment groups based on the order of inclusion, rather than through a true randomization process. This was adopted for convenience and to accommodate the dynamics of the outpatient clinic) was conducted between October 2018 and March 2020, at the outpatient clinic of the Hospital de Clínicas de Porto Alegre (HCPA) - RS, Brazil. The study enrolled 48 patients, who were sequentially assigned to undergo either vaginal laser treatment (24 patients) or vaginal promestriene treatment (24 patients). The project was submitted and approved by the Brazilian Registry of Clinical Trials (ReBEC, UTN U1111-1317-5796).

The criteria for inclusion were women in menopause (at least 12 months after their last menstruation, or bilateral oophorectomy or hysterectomy with follicle-stimulating hormones >40 U/L) and a recent negative Pap smear, who wanted to reduce symptoms of GSM, and who were willing to participate and sign the Informed Consent Form (ICF).

The criteria for exclusion were current or recent vaginal lesions (<15 days), use of HT within the last 3 months, untreated infections of the genitourinary tract, abnormal uterine bleeding, a history of photosensitivity disorder or the use of photosensitizing drugs, second or third degree genital prolapse according to the Pelvic Organ Prolapse Quantification System (POP-Q), bacterial or viral vaginal infection, immunosuppression, chronic use of corticosteroids, scleroderma, previous radiotherapy, burns in the area, collagen diseases, genital neoplasia, anticoagulant therapy, patients with a sling/mesh or uncontrolled diabetes.

At the first evaluation, the patient's eligibility was verified, using a checklist of the criteria for inclusion or exclusion. Sociodemographic and clinical information was obtained, and the women answered the FSFI-6 and the ICIQ-SF questionnaires. They also provided ratings on VAS for the three main symptoms of GSM (vaginal dryness, burning/irritation, and dyspareunia) on a scale from 0 to 10, where 0 indicated no symptom or discomfort and 10, the highest level of discomfort.

Next, the researcher and the gynecologist, carried out a speculum examination to evaluate the vaginal mucosa using the VHI, determining elasticity, amount of secretion, integrity of the epithelium, moisture, and pH using an Ecocare Comfort™ brand strip, assigning a score of 1 to 5 for each item. Subsequently, a local anesthetic of 2% lidocaine without a vasoconstrictor was applied and a punch biopsy (3 mm) was performed on the middle third of the right lateral wall of the vagina.

The biopsies specimens were sent to the Pathology Laboratory for hematoxylin and eosin (HE) staining. One trained pathologist examined all samples and was blinded to which group the patient belonged (CO_2_ laser or promestriene).

The histological slides were analyzed, and all observed alterations were described in comparison to the expected normal histological pattern. The evaluated parameters included average epithelial thickness and the presence of glycogen in the squamous epithelium. The histological criteria for normality included: absence of lymphocytes and neutrophils; abundant glycogen in the intermediate and superficial epithelial layers—serving as an indicator of adequate epithelial maturation, based on a qualitative assessment of the entire epithelium; epithelial thickness of approximately 150 µm;^(9)^ and absence of neovascularization in the biopsied areas. Blood vessels were counted per low-power field and stratified as follows: none; grade 1 (<3 vessels); grade 2 (3–5 vessels); and grade 3 (>5 vessels). Collagen fibers were readily identifiable and were quantified by a trained pathologist. The quality of the histological sections was also assessed based on the proper orientation of the tissue within the paraffin block and the parallel alignment of the basal membrane relative to the epithelial surface. To standardize the assessment of vaginal atrophy, a histological scoring system—designated the Vaginal Trophism Histologic Score (VTHS)—was developed to facilitate the structured presentation of findings (Table 1).

**Table 1:** Vaginal Trophism Histological Score (VTHS)

Parameters*	0	1	2	3	Total Score
Average epithelial thickness (100 x)	None or incomplete	Atrophy <150 µm	Hypertrophy>150 µm		
Stroma (collagen fibers)	None	Scarce	Moderate	Plenty	
Presence of glycogen	None	<20%	20-50%	>50%	
Presence of lymphocytes	None	<10	10-30	>30	
Presence of neutrophils	>10	5-10	<5	None	
Vascularization (No. of vessels)	None	<3	3-5	>5	
Quality of the slice	Inadequate		Adequate		

* This score was developed by the authors to standardize and quantify the histological parameters related to vaginal atrophy by hematoxylin and eosin (HE) staining

CO_2_ Laser Group underwent 3 sessions of vaginal CO_2_ pixel laser treatment (FemiLift, Alma Lasers, Buffalo Grove, IL, USA) with an interval of 30 days. The CO_2_ laser device was programmed for 80 mJ, 9×9 pixels, on medium power. After removing the excess secretion from the vagina, the probe was placed in a disposable cover lubricated with mineral oil and was inserted into the end of the vagina. Further, the device was activated at angles corresponding to even hours of the clock, pulling and rotating for every 1 cm until the black ring of the applicator emerged from the vagina. Subsequently, it was reprogrammed for 100 J, 9×9 pixels, on medium power and applied to the odd angles. In the end, patients received after-laser care instructions and were scheduled for the next treatment after 30 days. This group was evaluated before (day 1) and 30 days after the end of the treatment (day 120).

Considering that promestriene loses its effect shortly after discontinuation, the Promestriene group received 1 g of promestriene cream (Eurofarma S.A, Brazil) - equivalent to one full vaginal applicator - daily for 14 days, followed by twice-weekly applications for 3 months and 3 weeks, ending one week before the biopsy performed on day 120. At the first consultation, they received a prescription and the promestriene cream sufficient for 30 days and were instructed to return to the outpatient clinic every month to check their use of the medication and to receive more cream for the following month. As with the CO_2_ Laser Group, evaluations for this group were conducted on day 1 (prior to treatment) and 7 days after the end of the treatment period (day 120).

The sample size was estimated using the WinPepi software for Windows (version 11.63) based on the reference of "Sexual Satisfaction" described by Salvatore et al. (2015).^(10)^ After laser use and considering a sample power of 80%, with 20% losses and a significance level of 5%, the final sample size required to detect the difference in means (2 units) will be 48 women (24 for the L group and 24 for the P group).

The data were entered into the SPSS program, version 21.0 (SPSS Inc. Released 2009. PASW Statistics for Windows, Version 21.0. Chicago: SPSS Inc.). Descriptive statistics were obtained using measures of central tendency and dispersion for continuous variables (mean standard deviation or median and interquartile range) and absolute (n) and relative (n%) frequencies for categorized variables, with a prior assessment of the distributions were found. The Shapiro–Wilk normality test was applied for continuous variables. Bivariate comparisons between groups were conducted using the Student's t-test for independent samples or the Mann–Whitney test, for quantitative variables, where applicable. Pearson's chi-square or Fisher's exact tests were used to compare proportions. The generalized estimating equations (GEE) model was used to simultaneously compare between and within groups, complemented by the least significant difference (LSD) test. For all the analyses, the level of significance was set at 5%.

The project was referred to the Human Research Ethics Committee of the Hospital de Clínicas de Porto Alegre (CEP-HCPA 2.676.945) and was found to be in accordance with international standards regulating human research (Certificado de Apresentação de Apreciação Ética: 85227417.3.0000.532).

## Results

Forty-three women completed the treatment, 22 from CO_2_ Laser Group and 21 patients from promestriene group. There was no difference regarding the socio-demographic and clinical variables between the two groups at baseline. The average age, been pregnant twice and the average menopause age was similar in both groups. Thirteen percent of patients in CO_2_ Laser Group and 33% of those in promestriene group had tried some type of hormone replacement before. Ninety percent of patients in both groups were sexually active and the majority, 16 (72.7%) in CO_2_ Laser Group and 18 (85.7%) in promestriene group, lived with a partner. Of the 43 women in the study, 5 in CO_2_ Laser Group and 2 in promestriene group had a history of breast cancer. Fourteen patients in CO_2_ Laser Group (63.6%) and eight in promestriene group (38.1%) used medication that could affect their libido (Table 2).

**Table 2:** Characteristics of the sample group

Variables	CO_2_ Laser Group (n=22) n(%)	Promestriene Group (n=21) n(%)	p-value
Age (years), average±SD	54.8±6.8	54.9±9.1	0.973
Sexually active	20(90.9)	19(90.5)	1.000
Married/partner	16(72.7)	18(85.7)	0.457
Number of pregnancies, md (P25–P75)	2(1–3)	2(1.5–3.5)	0.353
Number of caesarian sections, md (P25–P75)	0(0–1.3)	0(0–0.5)	0.228
Number of births, md (P25–P75)	0(0–1.3)	2(0–3)	0.075
Number of abortions, md (P25–P75)	0(0–1)	0(0–0)	0.131
Age at menopause (years), average±SD	47.9±5.1	47.9±5.1	0.979
Length of menopause (years), md (P25–P75)	5(2–9)	4(3–8.5)	0.903
Previous hormonal therapy	3(13.6)	7(33.3)	0.162
Length of previous hormonal therapy (years), md (P25–P75)	1(1–3)	3(1–12)	0.267
Previous history of neoplasm	5(22.7)	2(9.5)	0.412
Medication that affects libido	14(63.6)	8(38.1)	0.171

The VAS for symptoms of GSM showed a significant reduction for the scores of each item in both groups (p<0.001). The degree to which they complained of vaginal dryness decreased from 8.1 to 3.3 in CO_2_ Laser Group and reduced by 4.8 points between the initial and the final evaluation. In promestriene group, the degree to which they complained of vaginal dryness decreased from 7.5 to 3.5 and reduced by 3.95 points (p<0.001). The sense of vaginal burning decreased from 5.2 to 1.9 in CO_2_ Laser Group (reduced by 3.36 points between the initial and final evaluation) and from 3.95 to 1.95 in promestriene group (reduced by 2 points). Dyspareunia decreased from 6.96 to 3.46 points in promestriene group (reduced by 3.5 points between the initial and final evaluation) and 5.76 to 2.57 points in promestriene group (-3.19 points). The sum of the total VAS score for the symptoms of GSM was 20.4 at the initial evaluation and 3.46 points at the final evaluation for CO_2_ Laser Group (-11.7, difference between the scores). In promestriene group, the sum was 17.2 and 8.09 (-9.14, difference between the initial and final evaluation). In the comparison between the groups, there was no significant difference, indicating equivalence between the two types of treatment (Table 3).

**Table 3:** Visual Analog Scale for GSM symptoms (VAS) and Vaginal Health Index Score (VHI)

Variables		CO_2_ Laser Group (n=22)	Promestriene Group (n=21)	p-value
		Mean±SD	Mean±SD	
**Visual Analog Scale for GSM symptoms (VAS) Over Time**				
Vaginal dryness				
	Initial	8.14±2.77	7.52±3.01	0.477
	End	3.32±3.33	3.57±2.89	0.786
	Difference (IC 95%)	-4.82 (-6.02–-3.61)	-3.95 (-5.19–-2.72)	0.518
	p-value	<0.001	<0.001	
Vaginal burning				
	Initial	5.27±4.41	3.95±4.18	0.302
	End	1.91±2.99	1.95±3.12	0.962
	Difference (IC 95%)	-3.36 (-4.82–-1.91)	-2.00 (-3.19–-0.8)	0.376
	p-value	<0.001	0.001	
Dyspareunia				
	Initial	6.96±3.61	5.76±4.31	0.315
	End	3.46±3.79	2.57±3.52	0.417
	Difference (IC 95%)	-3.50 (-4.94–-2.06)	-3.19 (-4.57–-1.80)	0.761
	p-value	<0.001	<0.001	
Total				
	Initial	20.4±9.20	17.2±8.94	0.247
	End	8.68±8.89	8.09±7.92	0.814
	Difference (IC 95%)	-11.7 (-14.9–-8.44)	-9.14 (-11.8–-6.53)	0.671
	p-value	<0.001	<0.001	
**Vaginal Health Index Score (VHI) Over Time**				
Elasticity				
	Initial	2.50±0.60	2.91±0.70	0.037
	End	3.68±0.72	3.76±0.44	0.649
	Difference (IC 95%)	1.18 (0.94–1.42)	0.86 (0.62–1.10)	0.061
	p-value	<0.001	<0.001	
Amount of secretion				
	Initial	2.05±0.84	2.38±0.92	0.203
	End	3.68±1.04	3.67±0.91	0.959
	Difference (IC 95%)	1.64 (1.27–2.01)	1.29 (0.89–1.69)	0.206
	p-value	<0.001	<0.001	
Vaginal pH				
	Initial	1.91±0.97	2.86±1.24	0.004
	End	2.77±1.23	3.81±1.17	0.004
	Difference (IC 95%)	0.86 (0.44–1.29)	0.95 (0.45–1.46)	0.791
	p-value	<0.001	<0.001	
Integrity of the epithelium				
	Initial	3.27±1.20	3.19±1.36	0.830
	End	4.41±0.80	4.52±0.51	0.563
	Difference (IC 95%)	1.14 (0.68–1.60)	1.33 (0.78–1.88)	0.590
	p-value	<0.001	<0.001	
Moisture				
	Initial	2.32±0.89	2.57±1.03	0.378
	End	3.91±1.07	3.91±1.04	0.989
	Difference (IC 95%)	1.59 (1.18–2.00)	1.33 (0.91–1.76)	0.393
	p-value	<0.001	<0.001	
Total				
	Initial	12.0±3.50	13.9±4.00	0.097
	End	18.5±4.32	19.7±3.32	0.289
	Difference (IC 95%)	6.41 (5.05–7.77)	5.76 (4.41–7.11)	0.508
	p-value	<0.001	<0.001	

The Vaginal Health Index Score (VHI) showed a significant increase (p<0.001) in all scores for both groups (Table 3). The overall VHI score in CO_2_ Laser Group increased from 12 to 18.5 (+ 6.41 points after treatment). The score for promestriene group also increased from 13.9 to 19.7 (+ 5.76 points after treatment). The elasticity of the vaginal epithelium in promestriene group had a higher initial score, compared with CO_2_ Laser Group, and the improvement in elasticity after treatment was less evident. The difference between the initial and final elasticity of CO_2_ Laser Group was 1.64 and in promestriene group was 0.8 (Table 3). The vaginal pH score in CO_2_ Laser Group was 1.91 initially (pH>6.1) and further, 2.77 (pH 5.1-6) with an increase of 0.86 points between the evaluations. Promestriene group had an initial pH score of 2.86 (pH 5.1-6) and a final pH score of 3.81 (pH 4.7-5), an improvement of 0.95 points over the period. As for FSFI, there was a significant increase in all scores in both groups in relation to the questions about desire, lubrication, and the total score. When comparing the groups, CO_2_ Laser Group showed a significantly higher increase in the scores for lubrication, orgasm, and satisfaction and the total score, than promestriene group. Notably, the participants in promestriene group had significantly higher scores for satisfaction and total scores, than those in CO_2_ Laser Group, comparing the initial questionnaires (Table 4).

**Table 4:** Female Sexual Function Index (FSFI) and International Consultation on Incontinence Questionnaire-Short Form (ICIQ-SF)

Variables		CO_2_ Laser Group (n=22)	Promestriene Group (n=21)	p-value
		Mean±SD	Mean±SD	
**Female Sexual Function Index (FSFI)**				
Desire				
	Initial	1.41±0.67	1.67±1.06	0.333
	End	2.32±1.04	2.24±1.30	0.820
	Difference (IC 95%)	0.91 (0.60– 1.22)	0.57 (0.23– 0.91)	0.076
	p-value	<0.001	0.001	
Excitation				
	Initial	1.64±1.00	2.24±1.34	0.088
	End	2.41±1.10	2.62±1.60	0.606
	Difference (IC 95%)	0.77 (0.38–1.17)	0.38 (-0.05–0.81)	0.096
	p-value	<0.001	0.079	
Lubrication				
	Initial	1.32±0.84	1.95±1.56	0.092
	End	3.23±1.69	2.91±1.76	0.531
	Difference (IC 95%)	1.91 (1.38–2.44)	0.95 (0.32–2.22)	0.005
	p-value	<0.001	0.003	
Orgasm				
	Initial	1.77±1.51	2.48±1.76	0.149
	End	2.91±1.72	2.52±2.04	0.493
	Difference (IC 95%)	1.14 (0.60–1.68)	0.05 (-0.34–0.43)	0.001
	p-value	<0.001	0.808	
Satisfaction				
	Initial	1.82±1.14	2.76±1.48	0.017
	End	3.09±1.51	3.33±1.56	0.596
	Difference (IC 95%)	1.27 (0.71–1.84)	0.57 (-0.04–1.18)	0.032
	p-value	<0.001	0.068	
Pain				
	Initial	1.59±1.33	2.24±1.76	0.165
	End	3.27±1.91	3.00±2.10	0.648
	Difference (IC 95%)	1.68 (0.95–2.41)	0.76 (-0.18–1.70)	0.130
	P	<0.001	0.113	
Total				
	Initial	9.55±5.23	13.3±7.47	0.049
	End	17.2±7.24	16.6±9.23	0.806
	Difference (IC 95%)	7.68 (5.44–9.92)	3.29 (0.40–6.17)	0.004
	p-value	<0.001	0.026	
**International Consultation on Incontinence Questionnaire-Short Form (ICIQ-SF)**				
Frequency of urinary incontinence				
	Initial	0.96±1.65	1.19±1.57	0.624
	End	0.23±0.87	0.76±1.37	0.121
	Difference (IC 95%)	-0.73 (-1.32–-0.13)	-0.43 (-0.68–-0.18)	0.362
	p-value	0.016	0.001	
Amount of leakage				
	Initial	1.00±1.72	1.52±1.89	0.331
	End	0.27±0.94	0.76±1.48	0.187
	Difference (IC 95%)	-0.73 (-1.37–-0.08)	-0.76 (-1.38–-0.14)	0.939
	p-value	0.027	0.016	
Overall impact of urinary incontinence				
	Initial	1.36±2.80	2.43±3.63	0.272
	End	0.32±1.49	1.29±2.61	0.129
	Difference (IC 95%)	-1.04 (-2.07–-0.02)	-1.14 (-1.90–-0.38)	0.881
	p-value	0.046	0.003	
Total score				
	Initial	3.32±5.99	5.14±6.51	0.329
	End	0.82±3.23	2.81±5.09	0.118
	Difference (IC 95%)	-2.50 (-4.70–-0.29)	-2.33 (-3.70–-0.97)	0.297
	p-value	0.026	0.001	

There was a significant reduction of all scores in the ICIQ-SF for both groups. The differences between the groups were not significant (Table 4). Nine of the 22 patients in CO_2_ Laser Group and 16 of the 21 patients in promestriene group reported urinary incontinence (UI). All patients answered the questionnaire and a score of 0 represented the absence of incontinence in the initial and final scores. No patients developed UI after treatment in either group. Eight of the 9 patients in CO_2_ Laser Group reported that the frequency and amount of leakage decreased. They also stated that there had been an improvement in how much the leakage interfered with their daily life. The total score of all items evaluated decreased from 3.32 to 0.82, reducing by 2.5 points on average (p<0.026), which was significant, even with a small number of patients. Fifteen of the 16 patients in promestriene group reported that the frequency and amount of leakage decreased. The improvement in how much the leakage interfered with their daily life and the total score reduced from 5.1 to 2.81 (p<0.001).

The histological evaluation using VTHS was carried out for 22 patients in CO_2_ Laser Group and 20 patients in promestriene group. One of the samples in promestriene group was discarded because the histological slice of the vaginal epithelium was not suitable for evaluation.

The histological samples were classified as adequate or not, according to the quality of the staining, the cut, and the amount of tissue included. In CO_2_ Laser Group, 81.8% of the samples were considered adequate for both the initial biopsy and the final one. The suitable slides in promestriene group were 70% and 75%, respectively. This difference in quality between the groups was not significant and did not impair the data analysis (Table 5).

**Table 5:** Histological Score (VTHS) Over Time

Variables		CO_2_ Laser Group (n=22)	Promestriene Group (n=20)	p-value
		Mean±SD	Mean±SD	
Stroma				
	Initial	2.14±1.21	2.30±1.13	0.642
	End	2.77±0.53	2.65±0.75	0.532
	Difference (IC 95%)	0.63 (0.19–1.08)	0.35 (-0.30–1.00)	0.497
	p	0.005	0.294	
Epithelial thickness				
	Initial	1.00±0.87	0.90±0.85	0.700
	End	1.59±0.79	1.50±0.83	0.711
	Difference (IC 95%)	0.59 (0.09–1.09)	0.60 (0.06–1.14)	0.889
	p	0.020	0.031	
Glycogen				
	Initial	1.14±0.71	1.00±0.86	0.567
	End	1.64±0.73	1.85±1.09	0.448
	Difference (IC 95%)	0.50 (0.09–0.91)	0.85 (0.33–1.37)	0.329
	p	0.018	0.001	
Lymphocytes				
	Initial	0.73±0.63	0.95±0.51	0.196
	End	0.91±0.29	1.00±0.00	0.138
	Difference (IC 95%)	0.18 (-0.09–0.45)	0.05 (-0.17–0.27)	0.433
	p	0.189	0.653	
Neutrophils				
	Initial	2.36±0.73	2.45±0.51	0.646
	End	2.91±0.29	2.85±0.37	0.557
	Difference (IC 95%)	0.55 (0.22–0.87)	0.40 (0.14–0.66)	0.479
	p	0.001	0.002	
Vascularization				
	Initial	0.96±0.79	0.90±0.45	0.775
	End	1.82±0.50	1.55±0.69	0.141
	Difference (IC 95%)	0.86 (0.46–1.27)	0.65 (0.36–0.94)	0.649
	P	<0.001	<0.001	
Sufficient quality of the slice				
	Initial	18 (81.8%)	14 (70.0%)	0.368
	End	18 (81.8%)	15 (75.0%)	0.591
	Difference (IC 95%)	0.00% (-25.2% – 25.2%)	5.00% (-20.8% –30.8%)	0.818
	P	1.000	0.704	
Total score				
	Initial	9.95±2.79	9.90±2.38	0.944
	End	13.3±2.16	12.9±2.47	0.595
	Difference (IC 95%)	3.32 (1.79– 4.84)	3.00 (1.40– 4.60)	0.778
	P	<0.001	<0.001	

**Figure 1:**
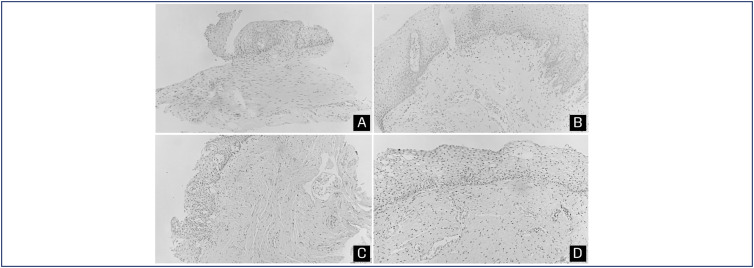
Histological Sections of the Vaginal Wall.

The appearance of the stroma considered the amount of collagen fibers and was classified as inadequate when these fibers were absent or scarce, and adequate, when they were in moderate numbers or abundant. There was an increase in the number of collagen fibers in both treatment groups between the initial and the final evaluation, but it was only significant (p>0.005) in CO_2_ Laser Group. This finding could be linked to the results of the elasticity evaluation of the vaginal epithelium using VHI, which was at a higher level in patients in promestriene group before treatment, compared with patients in CO_2_ Laser Group. The average epithelial thickness in CO_2_ Laser Group was 184.6 µm before treatment and 245 µm after. In promestriene group, the mean epithelial thickness was 150.7 µm before and 265.3 µm after treatment. A significant increase in epithelial thickness was observed for both CO_2_ Laser Group (p<0.02) and promestriene group (p<0.03). The amount of glycogen in the cells of the epithelium after treatment also increased and was significant for both CO_2_ Laser Group (p<0.01) and promestriene group (p<0.001). There was no significant change in the number of lymphocytes before and after treatment in both groups. However, there was a noticeable decrease in the number of neutrophils, indicating that there was less inflammatory atrophy in both groups, a significant reduction between the initial and final evaluations of CO_2_ Laser Group (p<0.001) and promestriene group (p<0.002). The vascularization of the epithelium, measured by the number of blood vessels, also demonstrated a significant increase for both groups (p<0.001). Regarding the histological score, there was an increase in the parameters such as epithelium thickness, glycogen, neutrophils, and vascularization for both groups (Figure 1). The initial score of CO_2_ Laser Group was 9.9, which increased to 13.3. The initial score of promestriene group was 9.9, which increased to 12.9; thus, the results for groups L and P were significant (p<0.001). There was no significant difference between the positive results of either treatment, which proved to be equivalent (Table 5).

## Discussion

In this study, a histological score to evaluate the degree of vaginal atrophy, named Vaginal Trophism Histologic Score (VTHS) was developed.

The comparison between vaginal CO_2_ laser therapy and promestriene for GSM is particularly relevant for patients who avoid hormonal treatments.^(11)^ While early studies suggested benefits,^(12)^ sham-controlled RCTs have shown limited efficacy for laser therapy.^(12,13)^ CO_2_ laser improves some symptoms, like daily dryness, but may worsen others. The lack of robust data and a clearly defined minimal clinically important difference (MCID) currently limits the clinical adoption of CO_2_ laser therapy.

The positive results of using the CO_2_ laser treatment observed in this study are similar to those featured in other studies.^(5,8,14-22)^ Although most of the articles published feature the CO_2_ scanner type laser (Monalisa Touch, DEKA laser, Florence, Italy) and not the pixel design (FemiLift, Alma Lasers, Buffalo Grove, IL, USA) which is featured in this study, the results from previous studies and this study are comparable. Tadir et al. published a review article in 2017 on the three most used types of laser and some of the radiofrequency devices. They concluded that each method produced a thickening of the vaginal epithelium, an increase in glycogen, remodeling of the collagen, new vascularization, and an increase in the amount of lactobacillus with reduced pH. Further, they found decreased vaginal wall diameter and improved urination with minimal risk of short-term and long-term complications.^(23)^

Most of the studies use the following subjective methods of assessing the effectiveness of the laser: VAS for symptoms such as vaginal burning, dyspareunia, and dryness; sexual functioning questionnaires such as FSFI; and urinary incontinence questionnaires such as UCIQ-SF. The results from the VAS were comparable in most studies.^(6)^ Similarly, the FSFI-6 and the urinary incontinence questionnaire showed significance in symptom improvement that was comparable to other studies.^(15,23-26)^

The most used objective methods for evaluating CO_2_ laser effectiveness are VHI, vaginal cytology, and pH measurement. Further, there was a demonstrable improvement in the indices relating to vaginal epithelium for all of these parameters, which reconfirms the existing data.^(15,27-29)^

Since 2018, prospective controlled articles began to be published, with longer follow-up time, a greater number of patients, and well-defined inclusion and exclusion criteria. Some of these authors compared the use of topical vaginal creams (estriol or promestriene) and vaginal laser (CO_2_ or erbium), in a design similar to this study, also demonstrating improvement in vaginal trophism both in the Laser Group and in the vaginal estrogen group.^(14-16,18-20,30-35)^

However, prospective controlled studies using histopathology of vaginal tissue before and after treatments were not found in the literature. Zerbinati et al.^(5)^ described the improvement in the vaginal mucosa in 5 patients, through a histopathological evaluation before and 2 months after using the CO_2_ laser, which demonstrated an increase in the thickness of the epithelium, presence of fibroblasts, and an increase in collagen and elastin.

Gaspar and colleagues studied 25 patients treated with topical estriol for 8 weeks, and 25 treated with topical estriol for 15 days before the application of a non-ablative erbium laser. Biopsies and histological evaluation of 6 patients in each group were performed before the start of the treatment and after 1, 3, 6, and 12 months. The results showed improvement in mucosal trophism, angiogenesis, congestion, and restructuring of the lamina propria. These changes were most pronounced in the Laser Group. The cell maturation index and the decrease in vaginal pH were also higher in the L group (p<0.05).^(14)^

Pagano et al. (2017) described 33 post-menopausal and oncological patients who underwent pixel CO_2_ laser (Femilift, Alma Lasers) treatment without a control group. They were evaluated using VHI, VAS, ICIQ-SF, and vaginal biopsy samples. The pixel CO_2_ laser induced neo-collagenogenesis, increased epithelial papillae thickness, and a significant enhancement of type III collagen production in the lamina propria after treatment.^(36)^ This study demonstrated a positive effect on vaginal atrophy through a histopathological examination of 42 patients, which identified an increase in the thickness of the vaginal wall, an improvement in tissue vascularization, an increase in the amount of cellular glycogen, and a decrease in neutrophils, both for the Laser Group and the promestriene group. In addition, the pixel CO_2_ Laser Group showed an increase in the number of collagen fibers, confirming the histological improvement of the vaginal wall. Conversely, Jaber et al. (2025),^(11)^ in a randomized, sham-controlled clinical trial involving breast cancer survivors with Genitourinary Syndrome of Menopause (GSM), reported mixed results, although some outcomes showed statistically significant improvements.

The difficulty of a SHAM group for the laser, the still restricted access to this technology in countries like Brazil, the difficulty of subjectively and objectively evaluating the effect of these treatments, and the long-term follow-up of these women continue to be the major cause of difficulties in research. Most studies focus on treatment satisfaction and sexual function of the patient, which are subjective and multifactorial variables. Subjective evaluations are not always in line with objective results. The most used objective variables are pH, VHI, and vaginal cytology. The collection of cytology at menopause can be hampered by the scarcity of material for analysis by the pathologist. The VHI also describes parameters that may vary, depending on the evaluator. Histological examination is an objective, visual and reliable parameter of vaginal trophism. Although it is simple to perform, a vaginal biopsy is still infrequent in larger studies, failing to collaborate in the objective comparison of the efficacy of treatments for GSM.

One limitation of this study was that the final number of participants did not reach the minimum sample size initially calculated to ensure adequate statistical power. This was due to follow-up losses and logistical challenges associated with patient recruitment in a specialized outpatient setting. Although the main outcomes showed statistically significant differences, the reduced sample size may have affected the interpretation of secondary outcomes and limited the generalizability of the results. We therefore recommend caution when extrapolating these findings and highlight the need for future studies with larger samples. Additionally, since patients were assigned to groups based on the order of inclusion, the non-randomized distribution represents another limitation of the study.

## Conclusion

This study highlights the viability of CO_2_ pixel laser is an effective treatment option for GSM, especially for women who cannot or do not wish to use hormonal therapies. The effect of the CO_2_ laser treatment was similar to the use of vaginal promestriene for all parameters evaluated in this study. However, further studies with larger samples and long-term follow-up are recommended to confirm these findings and evaluate the durability of the treatment effects. The inclusion of placebo groups and greater randomization can strengthen the evidence and provide a more comprehensive understanding of the benefits and limitations of CO_2_ pixel laser treatment for GSM.

## Data Availability

the authors did not make the data from this article available in repositories prior to submission.
